# Beyond productivity: Investigating keel bone fractures and welfare issues in the British dual-purpose breed Ixworth

**DOI:** 10.1016/j.psj.2025.106300

**Published:** 2025-12-16

**Authors:** Senta Becker, Wolfgang Büscher, Inga Tiemann

**Affiliations:** a Institute of Agricultural Engineering, University of Bonn, Nußallee 5, 53115 Bonn, Germany; b Chamber of Agriculture of North Rhine-Westphalia, Department of Animal Husbandry and Animal Breeding Law, Haus Düsse 2, 59505 Bad Sassendorf, Germany; c Faculty of Agricultural Sciences and Landscape Architecture, University of Applied Sciences Osnabrück, Emsweg 3, 49090 Osnabrück, Germany

**Keywords:** Animal welfare, Domestic chicken, Local breeds, Keel bone fractures, Welfare issues

## Abstract

This study explores welfare concerns, with a focus on keel bone fractures (KBF), in the traditional British breed Ixworth, a non-commercial dual-purpose chicken. Although keel bone fractures were lately discussed in the context of high laying performances, 69.2 % of hens exhibited KBF, particularly in the caudal region, challenging the notion that lower-performing breeds are less prone to this issue. The location of these fractures indicates that incomplete bone ossification may play a crucial role. Notably, we found that egg size during critical ossification age (31, 34 and 35 weeks of age (woa)), rather than total egg count, was the primary risk factor for KBF. Beyond KBF, other welfare indicators such as plumage condition and footpad lesions were evaluated. Plumage damage peaked at 50 woa but showed recovery towards the end of the laying period. The prevalence of footpad lesions, however, increased with age, reaching a peak at 70 woa. A moderate correlation between egg production and footpad lesions was identified, suggesting increased laying may exacerbate this issue. Our findings demonstrate that traditional breeds like the Ixworth might also show welfare problems, including KBF and footpad lesions. These results emphasize that reduced productivity does not necessarily mitigate welfare risks. Instead, risk factors like egg size and bone development should be considered in future research and breeding. Expanding this investigation to other breeds could further enhance our understanding of welfare challenges in alternative poultry systems.

## Introduction

In recent decades, animal welfare has gained significant importance. A recurring topic is that animals bred for lower performance levels may exhibit higher welfare standards (Tiemann et al., 2020). This is particularly relevant for traditional and local breeds, some of which are also suited as dual-purpose chickens. Studies involving various local breeds as well as the dual-purpose hybrid Lohmann dual have shown that their performance is lower compared to commercial layers, but compared to the latter, they show advantages in regard to welfare indicators such as plumage condition (Giersberg et al., 2017; Rieke et al., 2021; Schreiter and Freick, 2023) and footpad health (Lambertz et al., 2018; Schreiter and Freick, 2023). However, welfare issues still arise in these local breeds, especially regarding keel bone fractures (KBF) as a very prominent example (Jung et al., 2024).

In commercial laying hens, KBF are predominantly identified as one of the most significant welfare issues (Toscano et al., 2020), posing severe implications for the health and welfare of these birds (Riber and Hinrichsen, 2016). These fractures might not only cause pain and distress but can also lead to changes in behavior, reduced production performance and feed intake, causing economic losses in the poultry industry (Rufener et al., 2019; Wei et al., 2020). Hens with KBFs were also found to show depressive-like states (Armstrong et al., 2020). The etiology of KBF is described as multifactorial, involving genetic predispositions, housing conditions, nutritional deficiencies, and mechanical stressors (Toscano et al., 2020). Studies suggest that both external factors, such as husbandry design and perch provision, and internal factors, including bone strength and egg-laying processes, contribute to the prevalence of these fractures.

In general, selection for high laying performance may negatively influence bone health (Eusemann et al., 2020). Additionally, KBFs are rarely observed in roosters but are a prevalent problem in hens (Kittelsen et al., 2023). High-energy collisions seem not to be responsible for the majority of the fractures (Thøfner et al., 2020), indicating other underlying mechanisms. Abolishing egg production in commercial laying hens led to no appearances of KBFs (Eusemann et al., 2018), suggesting a direct relationship between laying performance and the presence of KBFs. However, KBFs do not only occur in high-performing modern laying hybrids. Non-commercial chicken breeds, often kept for hobby, exhibition, or conservation purposes, are also susceptible to KBF (Jung et al., 2024). Even in the ancestors of today's chickens, the red jungle fowl, the occurrence of KBF could be observed, however to a lesser amount and although these animals were never bred for show or performance (Kittelsen et al., 2020a, 2021). The fact that low-performing hens also show these fractures argues against high egg production as the exclusive factor. Variations in their prevalence have been found among different genotypes, indicating that selective breeding may help reduce the incidence (Kittelsen et al., 2020b). Understanding the prevalence of KBFs in different local breeds should raise awareness of welfare-associated problems even in lower-performing birds, but offers also interesting insights into etiology of KBF (Jung et al., 2024; Kittelsen et al., 2020b). Recent studies suggest that these might be caused by internal factors, supported by fractures appearing early in the laying period and especially at the caudal end of the keel bone. Modern laying hens in non-cage systems often show multiple fractures at the distal end of the keel bone with large callus formation (Malchow et al., 2022; Thøfner et al., 2020). These fractures may resemble greenstick fractures as described by Thøfner et al. (2020). In their study, Thøfner et al. (2020) also describe a mechanism causing fractures due to internal egg laying processes indicated by a positive association between KBF and the early age of the first egg, as well as the estimated egg weight at the onset of lay (Thøfner et al., 2021). Hens showing KBFs laid their first egg earlier, though there is no correlation with the total number of eggs (Gebhardt-Henrich and Fröhlich, 2015). The keel bone ossifies from the cranial to the caudal part, with ossification not finished until 35 weeks of age (woa), making it susceptible to damages, including fractures (Buckner et al., 1948). Recent radiographic (Gretarsson et al., 2024) and histological (Gretarsson et al., 2025) characterization confirm the late ossification in modern commercial laying hens, also describing great variability between individuals. The critical weeks of life in which individuals are susceptible for suffering KBF are therefore the weeks in which the ossification process is almost completed, while at the same time calcium resources are already needed for eggshell production (Wei et al., 2020).

Our research aligns with the first two critical research areas identified by Toscano et al. (2020), linking egg production and keel bone fractures and examining a non-commercial, local breed for their welfare status and keel bone health. This study aims to identify the patterns of welfare issues and the occurrence of KBF in the local dual-purpose breed British Ixworth and the correlation of these parameters to performance on an individual level. By analyzing individual performance and welfare data, risk factors for KBF were to be identified. Detailed pre- and post-mortem keel bone examinations were conducted to expand the current understanding of KBF.

## Materials and methods

### Ethics statement

The birds were housed at the Frankenforst Campus of the Agricultural Faculty, University of Bonn (53639 Königswinter, Germany). Animal husbandry was conducted in accordance with the regulations on animal protection and the keeping of production animals (Tierschutz-Nutztierhaltungsverordnung, 2006 (last revision 2021)) and the principles and specific guidelines presented in Guide for the Care and Use of Agricultural Animals in Research and Teaching (4th edition, 2020). The study was approved by the Animal Welfare Officer and the responsible committee of the Agricultural Faculty at the University of Bonn (Az 03.20.02_2020.11).

### Animals and husbandry conditions

The animals were hatched and reared as described in Becker et al. (2023) on the trial farm Campus Frankenforst of the Agricultural Faculty, University Bonn (53639 Königswinter, Germany; reg.no 39600305-547/17). By the first day of age, animals were marked individually with leg bands and later wing bands. The hens were kept in two separate groups, which differed in their feeding regime during the first ten weeks of rearing, but showed no differences in the welfare parameters examined (all *p* > 0.05). Focus was therefore set on individual data. The hens were divided into two groups: Group 1 consisted of 41 hens and 6 cockerels for breeding purposes. The 2nd group consisted of 35 hens and 6 cockerels. During the laying period, the animals were fed *ad libitum* with a commercial layer mash (all-mash LH; Deutsche Tiernahrung Cremer GmbH & Co KG, Düsseldorf, Germany; 17.5 % crude protein, 0.42 % methionine, 0.84 % lysine, 3.8 % calcium, 0.5 % phosphorus, 11.6 MJ ME/kg, without cocc.). Pen size for each group was 17.6 m^2^ (4 m x 4.4 m) in a conventional floor housing system corresponding to a stocking density of 2.33 animals m^−2^ for group 1 and 1.98 animals m^−2^ for group 2. The lighting program provided a 16-hour light and an 8-hour dark period (16L:8D). In addition, the birds were allowed daily access to a free-range outdoor area (108 m^2^ each). The pens were equipped with round feed dispensers and nipple drinkers as well as wooden perches, laying nests, dust bath, alfalfa bale and a pecking stone. The perches were square-shaped with rounded edges measuring 10 × 10 cm, and positioned at different heights (35, 65, and 95 cm above the floor).

The laying performance was monitored over one laying period, from the 20th to the 72nd week of life, at both group and individual bird levels. Individual trap nests (placed at two heights: 35 cm & 90 cm, each nest box measuring W x D x H: 28 × 46 × 55 cm) were used for this purpose. After an egg was laid, the hen was identified, and the egg was assigned to her. The egg's weight was also recorded. From Monday to Friday, eggs, their weights, and nest acceptance were recorded individually for each hen. On weekends, the trap nests were deactivated, and laying performance was recorded at the pen level. As a result, 54.8 % of the eggs were assigned to individual hens, while unassignable eggs, including those collected on weekends or holidays, were proportionally distributed among all hens and included in the calculations.

### Assessment of welfare parameters

In the 30th, 50th and 70th week of life, all birds of the flock were assessed for their welfare parameters according to the Welfare Quality® Assessment protocol for laying hens (WQA; Welfare Quality®, 2019). Focus was set on plumage damages, footpad lesions and keel bone damages. The parameters were classified in scores ranging from 0 to 2 as shown in Table 1.

**Table 1:** Underlying scores of the welfare assessment addressing plumage damage, footpad lesions and keel bone damages (Welfare Quality® Assessment protocol for laying hens).

Score	Plumage Damages
0	No or slight wear, (nearly) complete feathering (only single feathers lacking)
1	Moderate wear, i.e. damaged feathers (worn, deformed) or one or more featherless areas < 2.5 cm in diameter at the largest extent
2	At least one featherless area ≥ 2.5 cm in diameter at the largest extent

Footpad lesions	
0	Feet intact, no or minimal proliferation of epithelium, no wounds
1	Necrosis or proliferation of epithelium or chronic bumble foot with no or moderate swelling, not dorsally visible
2	Swollen (dorsally visible)

Keel bone damages	
0	No deviations, deformations or thickened sections, keel bone completely straight
1	Deviations (flattening, s-shape, bending) or thickened sections present in very slight form
2	Deviation or deformation of keel bone (including thickened sections)

### Further examination of Keel bone damages

As the palpation method does not provide accurate results according to previous studies, the keel bones were dissected after slaughter at 104 woa. The sample size was reduced because the wing bands of some carcasses were lost during mechanical plucking. Only identifiable carcasses were considered for further investigation (*N* = 52). Dissected keel bones were then skeletonized by beetles of the species *Dermestes vulpinus* so that the bones were cleaned of flesh after approx. 2-3 weeks and could be examined further (Meeuse, 1965). This method was chosen to avoid any manual manipulation of the bones. The exposed bones were then measured and examined for fractures. The entire length of the keel bone was measured, the number of fractures counted, and their position in relation to the total length of the bone determined.

### Statistical analysis

The statistical evaluation was carried out with the program SPSS Statistics 28 (IBM Corporation, Armonk, NY, USA). The significance level α was set at *p* ≤ 0.05 being significant, *p* ≤ 0.01 very significant and *p* ≤ 0.001 as highly significant. Comprehensive individual-specific performance data were collected for the animals considered in this study, as reported in detail in Becker et al. (2023). In contrast to earlier work, the underlying individual performance data was calculated in a novel way using the recorded welfare parameters. Correlations were used to check for interactions between performance and welfare parameters as well as keel bone damages and individual average egg weight during the specified weeks of age (up until 35 woa, the time where ossification is described to be finished (Buckner et al., 1948)). To calculate the correlation, the Shapiro-Wilk test was used first to test the data for normal distribution. For normally distributed data, the Pearson correlation coefficient was used. For non-normal distributed data, the Spearman-Rho correlation with the correlation coefficient R being set at |R|= .10 as low correlation, for |R|= .30 as moderate correlation and at |R|= .50 as high correlation was used (Cohen, 1988).

## Results

### Assessment of welfare parameters throughout the laying period

**Fig. 1:**
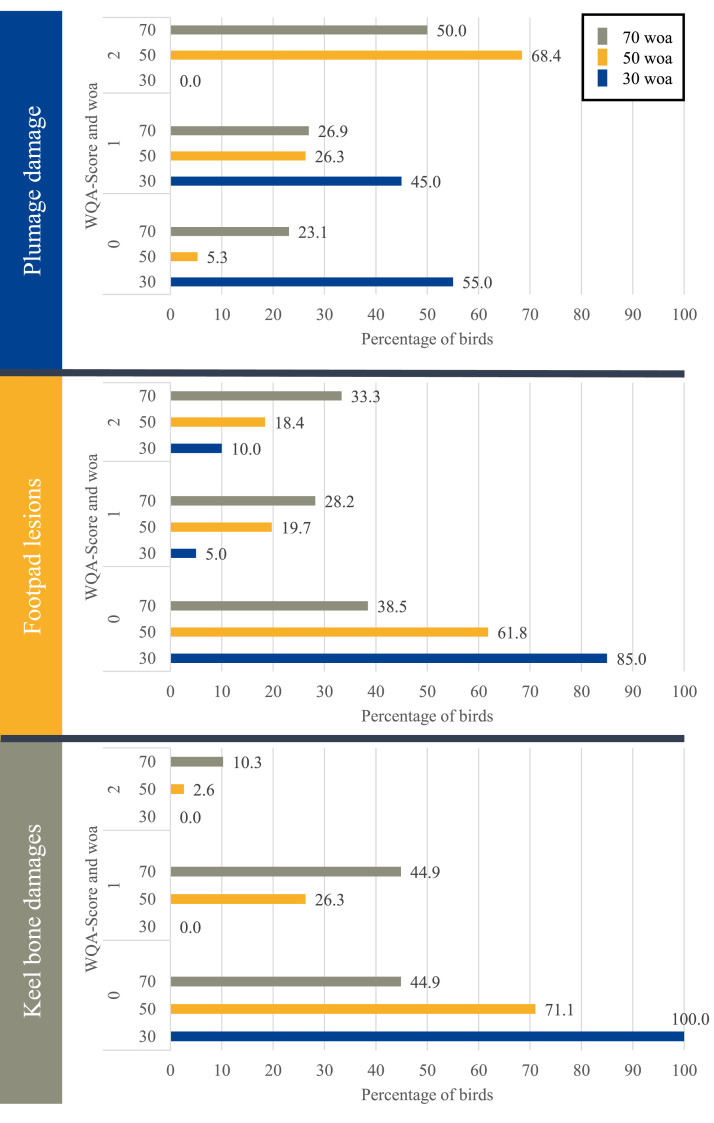
Distribution of welfare issues in Ixworth hens in correlation to age. The bar shows the percentage of animals affected by feather damage, footpad lesions, and keel bone damages based on a Welfare Quality® Assessment protocol for laying hens (WQA) assessment scale (0-2) in correlation to the age at assessment (30, 50, and 70 weeks of age (woa)).

The results for welfare assessment given in terms of the percentage of animals corresponding to each score at each time point of assessment are presented in Fig. 1. Regarding plumage damage, it was observed that the condition was worse at 50 woa compared to 70 woa indicated by a higher prevalence of score 2. Plumage damage in general was already present at 30 woa, given by 45.0 % with score 1. Towards the end of the laying period, plumage damage decreased to 50.0 % in score 2.

In the 30th week of age, the majority of animals exhibited no footpad lesions. However, the incidence of footpad lesions increased as the laying period progressed, with the highest scores appearing at 70 woa (33.3 % with score 2).

Regarding keel bone damage, no abnormalities were observed through palpation at 30 woa. With increasing age, the prevalence of KBF increased to a maximum of 10.3 % of the animals receiving a score of 2, and almost half of the animals receiving a score of 1.

### Correlation of welfare parameters and performance

Based on individual data, correlations between welfare scores and the performance parameters of body weight, onset of lay, average egg weight, and total egg count were calculated. The correlations of each parameter are presented in Table 2.

**Table 2:** Coefficients of correlations among egg production traits and welfare parameters.

Traits	Body weight [g]	Total egg count [n]	Ø Egg weight [g]	Onset of lay [d]
Plumage damages	−0.025	0.016	0.204	−0.219
Footpad lesions	−0.058	0.257 *	−0.100	−0.087
Keel bone damages	−0.095	0.170	0.172	−0.170

* p ≤ 0.05, ** p < 0.01 and *** p < 0.001.

No significant correlation was found between plumage damage and the performance parameters under consideration. Regarding footpad lesions, no correlation was observed with body weight, onset of lay, or average egg weight. However, a moderate positive correlation was found for scores of footpad lesions with the total number of eggs laid (*n* = 76; *r* = 0.257; *p* = 0.025). This positive correlation indicates that individuals laying more eggs are more likely to experience higher footpad scores i.e., lower footpad health. When analyzing keel bone scores gained by palpation, no correlations were found between scores and the performance parameters.

### Incidence and distribution of keel bone fractures

The keel bone was intensively examined, after being cleaned by *Dermestes vulpinus.* The keel bones were measured regarding their length, the prevalence of fractures and their position. In total, 69.2 % of the animals showed at least one fracture along the keel bone. Overall, 30.8 % of the birds showed no fractures, 25.0 % had one fracture, and 23.1 % of the birds exhibited two fractures. In 13.5 % of the birds, three fractures were detected, while 7.7 % presented the maximum number of four fractures.

**Fig. 2:**
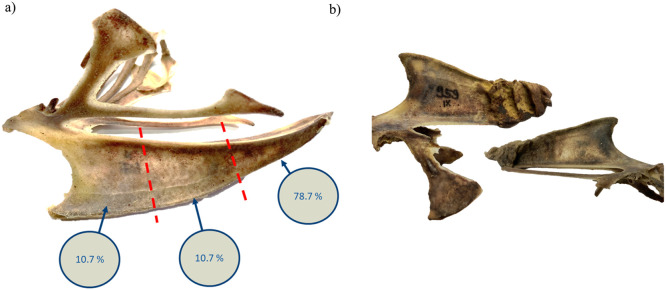
Distribution of keel bone fractures (KBF) in laying Ixworth hens. The left image (a) highlights the prevalence of KBF in the three sections along the keel bone (cranial, medial and caudal), with the corresponding percentages of fractures observed at each location. The right image (b) shows examples of fractured keel bones with multiple fractures located in the caudal part of the keel bone.

Fractures were predominantly found in the caudal part of the keel bone. Specifically, 10.7 % of the fractures were located in the cranial and medial part of the keel bone, respectively, while 78.7 % of the fractures were located in the caudal part (see Fig. 2a). It was also notable that the majority of keel bones of animals with multiple fractures exhibited an accordion-like structure (87 %). The keel bone appeared compressed, with several fractures aligned in sequence (see Fig. 2b).

### Correlation between egg weight and keel bone fractures in critical weeks

**Fig. 3:**
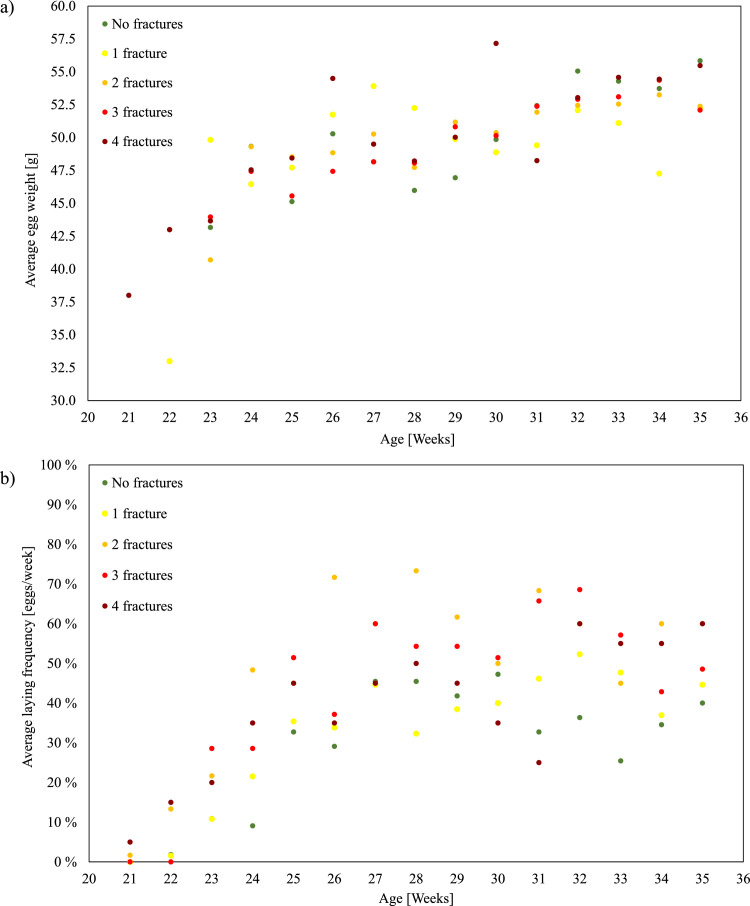
(a) Average egg weight (g) across the recorded weeks of life. Each point represents the mean value for the group of hens categorized by the number of keel bone fractures detected (0-4), indicated by point color. (b) Average weekly laying frequency (%) for the same hen groups with color points again corresponding to the number of keel bone fractures.

Since the fractures primarily occurred in the caudal part of the keel bone, and no correlation could be found between performance parameters (live weight, total egg count, average egg weight, and onset of lay) and their occurrence, additionally, for each bird, the egg size laid during each woa was analyzed in the context of *post mortem* KBF. Fig. 3 illustrates the average egg weight and laying frequency across age groups in relation to the number of keel bone fractures detected.

**Fig. 4:**
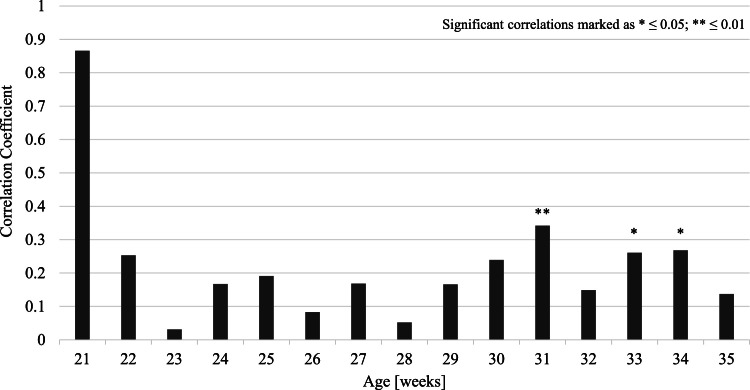
Correlation between keel bone damage at the end of laying period (post-mortem) and average egg weight in the respective weeks of age. The bar chart illustrates the value of correlation coefficients. Significant correlations are marked as * (p ≤ 0.05) and ** (p ≤ 0.01).

For each age, between the onset of lay and 35 woa (when the ossification of the keel bone has been found to be completed), a correlation was calculated comparing the average egg weight of the given week with the final keel bone score found after post-mortem examination. Moderate positive correlations were evident in weeks 31, 34, and 35 (see Fig. 4). These findings suggest that the higher the egg weight during these critical weeks, the higher the risk of keel bone fractures. The high correlation coefficient observed in week 21 is negligible, as only two birds were included in this analysis.

## Discussion

This study offers novel insights into the prevalence and characteristics of welfare issues and especially KBF in the traditional dual-purpose breed British Ixworth. The findings contribute to the growing body of research on welfare issues in non-commercial laying hens, as these are poorly investigated yet (Jung et al., 2024; Schreiter and Freick, 2023). Although here, the prevalence of KBF was found lower than in high-performing commercial breeds where up to 100 % individuals within a flocks can be affected (Thøfner et al., 2020), a significant percentage of hens (69.2 %) still exhibited fractures, particularly in the caudal region of the keel bone. Overall, 44.3 % even exhibited more than one KBF, which is also seen in commercial strains (Kittelsen et al., 2021). This challenges previous assumptions that lower-performing breeds inherently face fewer welfare issues, as previously suggested in earlier studies (Jung et al., 2024; Kittelsen et al., 2020b; Rieke et al., 2021; Schreiter and Freick, 2023). Our data imply that local breeds still remain vulnerable to KBF, indicating that factors other than general overall laying performance contribute to the occurrence of these fractures. Eusemann et al. (2018) found that suppressing egg production via hormonal treatment between week 24 to 35 completely prevented the occurrence of KBF, whereas up to 40 % of untreated hens exhibited KBF, emphasizing that physiological processes related to egg-laying might play a more significant role than previously understood.

In line with other findings (Malchow et al., 2022; Thøfner et al., 2020), the fractures found in the Ixworth hens were predominantly located in the caudal part of the keel bone. These fractures were described as greenstick fractures (Thøfner et al., 2020), a type of incomplete fracture where the bone bends and cracks rather than breaking completely. Greenstick fractures occur when the bone is still partially flexible, particularly during the early stages of ossification (Rodríguez-Merchán, 2005). The keel bone of laying hens ossifies gradually from cranial to caudal, with full ossification occurring by approx. 35 woa (Buckner et al., 1948; Gretarsson et al., 2024, 2025). Although the exact mechanisms leading to KBF are still not fully understood (Toscano et al., 2020), Thøfner et al. (2021) reported a positive correlation between KBF and early age at first egg and the estimated daily egg weight at the onset of lay. Similarly, Gebhardt-Henrich and Fröhlich (2015) noted that hens with KBF laid their first eggs earlier. This recurring observation may support the hypothesis that mechanical forces exerted during egg-laying, particularly in the early weeks of laying, may contribute to fractures. Here we identified the critical weeks during which our data point out a correlation between average egg weight and KBF (weeks 31, 34, and 35) which is congruent with the later stages of keel bone ossification. This suggests that heavier egg production during this vulnerable period could increase stress on the still-ossifying keel bone, leading to fractures.

In our study, the absence of a correlation between the total number of eggs laid and the occurrence of KBF is consistent with the findings of Gebhardt-Henrich and Fröhlich (2015) suggesting that the frequency of egg-laying may not be an significant risk factor as previously thought. Instead, our results highlight the relevance of production characteristics during the early laying period, especially the importance of egg size. Thøfner et al. (2021) identified the estimated relative daily egg weight (a metric integrating both laying rate and total egg mass) as a relevant predictor for KBF. This metric implies that lower individual laying rates with larger egg sizes may also influence the prevalence of KBF. Consequently, our findings emphasize the need for future studies not to focus on exclusively overall laying performance but on specific production metrics, such as egg size at certain stages of the laying period, when assessing welfare risks in laying hens. Individual data collection will be the key factor in understanding the underlying processes leading to such welfare issues (Becker et al., 2023).

In addition to KBF, this study also assessed other key welfare indicators, specifically plumage condition and footpad lesions, which are critical to the evaluation of the overall welfare status of hens. However, significant correlations between these welfare parameters (plumage damage, footpad lesions) and performance traits (body weight, onset of lay, total egg count) were to a large extend lacking.

Our results showed that plumage damage was most severe at 50 woa, with improvement towards the end of the laying period, which contrasts other studies in which plumage condition typically decreased over time (Giersberg et al., 2017). The unique social structure in our study, with small groups of hens and roosters, likely influenced these findings. Roosters contributed during the reproduction process to broken and torn feathers early on, particularly in the back region, complicating the distinction between damage from reproduction and potential feather pecking. However, the observed improvement toward the end of the period may reflect a stabilization of social dynamics or environmental adaptations (e.g., early moulting).

Footpad lesions increased progressively within the observation period which is concerning given the negative impact of such lesions on overall welfare (Lay et al., 2011). Similar results were observed in broiler breeders, which are comparable in size and weight to the Ixworth. Here, too, decreasing footpad health was found with increasing age (Kittelsen et al., 2024). As with FDP, also the risk of systemic bacterial increases with age decreasing overall welfare as shown for broiler breeders (Thøfner et al., 2019).

Interestingly, we found a moderate positive correlation between the total number of eggs laid and the severity of footpad lesions. This result aligns with Lambertz et al. (2018), who found similar patterns in other non-commercial breeds. However, this relationship requires further investigation of the underlying physiological mechanisms. As with plumage damage, footpad health is influenced by a combination of factors including litter quality, moisture, and social structures (Heerkens et al., 2016; Lay et al., 2011). In addition, welfare issues are also affected by individual differences (Schürmann et al., 2023). Even though dual-purpose and traditional breeds are often reported to have better plumage and footpad health (Giersberg et al., 2017; Schreiter and Freick, 2023), they remain susceptible to these welfare issues (Lambertz et al., 2018).

These results also raise important questions about the broader implications for non-commercial chicken breeds. While alternative breeds, such as the Ixworth, are expected to be more robust and resilient to welfare issues due to their lower productivity, our findings suggest that welfare issues, especially KBF, are also relevant to these genotypes (Jung et al., 2024). So far, selective breeding for higher egg production was thought to be the primary cause of fractures as well as other welfare issues (Eusemann et al., 2020). Instead, it appears that other factors, such as skeletal development, play crucial roles in determining the susceptibility of hens to KBF.

The lack of accurate diagnostic tools for detecting KBF in living hens remains a significant limitation in welfare assessment in both commercial and non-commercial poultry production. While palpation is commonly used, studies have shown a poor diagnostic accuracy (Kittelsen et al., 2023). Radiography and CT scans offer more precise diagnoses, but are costly and time-consuming (Toscano et al., 2020). Developing more cost-effective, non-invasive diagnostic techniques could greatly improve the ability to monitor and address KBF in real-time.

However, selecting for high egg-laying performance can negatively impact keel bone health (Eusemann et al., 2020). Our findings suggest that it is not the total number of eggs laid, but specific production characteristics (e.g. egg size, individual laying frequency) during critical weeks of bone ossification that play a more significant role in the development of keel bone fractures. The production of larger eggs early in lay as well as lower individual laying frequency may also increase susceptibility to fractures. The early onset of laying, driven by genetic selection for early laying maturity, contributes to an earlier increase in egg size, potentially increasing the risk of fractures. Extending the period of smaller egg production, while also supporting a regular laying rate with reduced variation in egg size, beyond the critical weeks of keel ossification may offer a promising solution. This could be achieved by revising breeding strategies to prioritize a biologically adapted variation in egg sizes during the laying period. While such changes could improve hen welfare by reducing the incidence of keel bone fractures, they might also lead to economic trade-offs, such as reduced early egg yield. Balancing welfare improvements with economic viability will require collaboration between breeders, producers, and researchers to develop sustainable solutions. The conservation of alternative poultry breeds is essential, as they represent valuable genetic resources for future breeding programs (Kittelsen et al., 2020b; Schreiter and Freick, 2023).

The relevance of these findings extends beyond the specific breed examined in this study. Damage to the keel bone is considered a major animal welfare problem in laying hen housing systems worldwide (Toscano et al., 2020) and affects both conventional high-performance hybrids as well as alternative and dual-purpose genotypes (Jung et al., 2024; Kittelsen et al., 2020a, 2021; Thøfner et al., 2021). As animal welfare and health play an increasingly important role alongside high performance, understanding these risk factors, especially in the early stages of life is becoming increasingly important for later ages. Incorporating such traits into breeding goals and management strategies can therefore contribute to improving animal welfare in a variety of international production systems.

To address the issue of keel bone fractures, further research could also investigate calcium balance in laying hens. Specifically, studies should quantify how much calcium is absorbed through feed, how much is utilized for egg production, and how much contributes to bone development. Previous research related to osteoporosis and so-called cage layer fatigue has already highlighted that calcium dynamics can vary across genetics, environment and nutrition, but comprehensive understanding is still lacking (Fleming et al., 2006; Webster, 2004). Such analyses could be conducted across different genetic lines and age groups to capture variations in calcium dynamics during critical growth and laying phases. Future studies should also examine possible influences of mating behavior in mixed-sex groups.

One limitation of this study is the relatively small sample size, particularly the reduced number of carcasses due to lost wing bands during mechanical plucking. While the results on an individual level provide valuable insights, a larger sample size could improve the robustness of the findings. Additionally, this study only focused on one traditional breed, the British Ixworth. Future research should aim to investigate a wider range of non-commercial and dual-purpose breeds to determine whether the patterns observed here are consistent across different genetic backgrounds.

In conclusion, this study expands the understanding of KBF in non-commercial laying hens, particularly in the British Ixworth breed. Our results based on animal-specific individual data reveal that even lower-performing breeds are vulnerable to welfare issues, including KBF, especially during critical stages of keel bone development. While it was not the total number of eggs laid, but production characteristics including egg size and individual laying rate during early lay, that emerged as a significant risk factor for fractures, the findings underscore the complex interactions between performance metrics and skeletal health. Future research should aim to refine welfare assessments by incorporating individual-level data and exploring non-invasive diagnostic tools for detecting KBF. Additionally, continued investigation into the genetic and physiological factors that influence bone health and especially ossification is crucial for developing strategies to improve the welfare of both commercial and alternative poultry breeds.

## Funding

This research was funded by the Ministry for Agriculture and Consumer Protection of the state of North Rhine-Westphalia, grant number 17-06.02.01 – 08/2020.

## CRediT authorship contribution statement

**Senta Becker:** Conceptualization, Data curation, Formal analysis, Funding acquisition, Investigation, Methodology, Project administration, Validation, Visualization, Writing – original draft, Writing – review & editing. **Wolfgang Büscher:** Project administration, Resources, Supervision, Writing – review & editing. **Inga Tiemann:** Conceptualization, Funding acquisition, Methodology, Project administration, Resources, Supervision, Visualization, Writing – original draft, Writing – review & editing.

## Disclosures

The authors declare the following financial interests/personal relationships which may be considered as potential competing interests:

Senta Becker reports financial support was provided by Ministry for Agriculture and Consumer Protection of the state of North Rhine-Westphalia. If there are other authors, they declare that they have no known competing financial interests or personal relationships that could have appeared to influence the work reported in this paper.

## References

[bib0001] Armstrong E.A., Rufener C., Toscano M.J., Eastham J.E., Guy J.H., Sandilands V., Boswell T., Smulders T.V. (2020). Keel bone fractures induce a depressive-like state in laying hens. Sci. Rep..

[bib0002] Becker S., Büscher W., Tiemann I. (2023). The British Ixworth: individual growth and egg production of a purebred dual-purpose chicken. Br. Poult. Sci..

[bib0003] Buckner G.D., Insko W.M., Henry A.H., Wachs E.F. (1948). Rate of growth and calcification of the sternum of male and female New Hampshire chickens. Poult. Sci..

[bib0004] Cohen J. (1988).

[bib0005] Eusemann B.K., Patt A., Schrader L., Weigend S., Thöne-Reineke C., Petow S. (2020). The role of egg production in the etiology of keel bone damage in laying hens. Front. Vet. Sci..

[bib0006] Eusemann B.K., Sharifi A.R., Patt A., Reinhard A.-K., Schrader L., Thöne-Reineke C., Petow S. (2018). Influence of a sustained release deslorelin acetate implant on reproductive physiology and associated traits in laying hens. Front. Physiol..

[bib0007] Fleming r.H., McCormack H.A., McTeir L., Whitehead C.C. (2006). Relationships between genetic, environmental and nutritional factors influencing osteoporosis in laying hens. Br. Poult. Sci..

[bib0008] Gebhardt-Henrich S.G., Fröhlich E.K.F. (2015). Early onset of laying and bumblefoot favor Keel bone fractures. Animals.

[bib0009] Giersberg M.F., Spindler B., Kemper N. (2017). Assessment of plumage and integument condition in dual-purpose breeds and conventional layers. Animals.

[bib0010] Gretarsson P., Kittelsen K., Moe R.O., Toftaker I., Thøfner I. (2025). Histomorphological characteristics of keel bone maturation and keel bone fractures in laying hens. Avian Pathol..

[bib0011] Gretarsson P., Søvik Å., Thøfner I., Moe R.O., Toftaker I., Kittelsen K. (2024). Fracture morphology and ossification process of the keel bone in modern laying hens based on radiographic imaging. PLoS ONE.

[bib0012] Heerkens J.L.T., Delezie E., Ampe B., Rodenburg T.B., Tuyttens F.A.M. (2016). Ramps and hybrid effects on keel bone and foot pad disorders in modified aviaries for laying hens. Poult. Sci..

[bib0013] Jung L., Hillemacher S., Tiemann I., Lepke M., Hinrichs D. (2024). Presence of keel bone damage in laying hens, pullets and roosters of local chicken breeds. PLoS ONE.

[bib0014] Kittelsen K.E., Gretarsson P., Jensen P., Christensen J.P., Toftaker I., Moe R.O., Vasdal G. (2021). Keel bone fractures are more prevalent in White Leghorn hens than in Red Jungle fowl hens-A pilot study. PLoS ONE.

[bib0015] Kittelsen K.E., Jensen P., Christensen J.P., Toftaker I., Moe R.O., Vasdal G. (2020). Prevalence of keel bone damage in red jungle fowls (Gallus gallus)-A pilot study. Animals.

[bib0016] Kittelsen K.E., Moe R.O., Hansen T.B., Toftaker I., Christensen J.P., Vasdal G. (2020). A descriptive study of keel bone fractures in hens and roosters from four non-commercial laying breeds housed in furnished cages. Animals.

[bib0017] Kittelsen K.E., Toftaker I., Tahamtani F., Moe R.O., Thøfner I., Vasdal G. (2023). Keel bone fractures in broiler breeders: is palpation a reliable diagnostic method?. Avian Pathol.

[bib0018] Kittelsen K.E., Vasdal G., Thøfner I., Tahamtani F. (2024). A walk through the broiler breeder life: how do footpad dermatitis and gait scores develop from rearing to slaughter?. Avian Pathol..

[bib0019] Lambertz C., Wuthijaree K., Gauly M. (2018). Performance, behavior, and health of male broilers and laying hens of 2 dual-purpose chicken genotypes. Poult. Sci..

[bib0020] Lay D.C., Fulton R.M., Hester P.Y., Karcher D.M., Kjaer J.B., Mench J.A., Mullens B.A., Newberry R.C., Nicol C.J., O'Sullivan N.P., Porter R.E. (2011). Hen welfare in different housing systems. Poult. Sci..

[bib0021] Malchow J., Eusemann B.K., Petow S., Krause E.T., Schrader L. (2022). Productive performance, perching behavior, keel bone and other health aspects in dual-purpose compared to conventional laying hens. Poult. Sci..

[bib0022] Meeuse A. (1965). The cleaning of skeletons by means of larvae of Dermestid beetles. Bijdragen Tot De Dierkunde.

[bib0023] Riber A.B., Hinrichsen L.K. (2016). Keel-bone damage and foot injuries in commercial laying hens in Denmark. Anim. Welf..

[bib0024] Rieke L., Spindler B., Zylka I., Kemper N., Giersberg M.F. (2021). Pecking behavior in conventional layer hybrids and dual-purpose hens throughout the laying period. Front. Vet. Sci..

[bib0025] Rodríguez-Merchán E.C. (2005). Pediatric skeletal trauma. Clin. Orthop. Rel. Res..

[bib0026] Rufener C., Baur S., Stratmann A., Toscano M.J. (2019). Keel bone fractures affect egg laying performance but not egg quality in laying hens housed in a commercial aviary system. Poult. Sci..

[bib0027] Schreiter R., Freick M. (2023). Laying performance characteristics, egg quality, and integument condition of Saxonian chickens and German Langshan bantams in a free-range system. J. Appl. Poult. Res..

[bib0028] Schürmann P., Becker S., Krause E.T., Hillemacher S., Büscher W., Tiemann I. (2023). Exploratory study on individual locomotor activity in local dual-purpose and commercial breeder pullets. Animals.

[bib0029] Thøfner I., Hougen H.P., Villa C., Lynnerup N., Christensen J.P. (2020). Pathological characterization of keel bone fractures in laying hens does not support external trauma as the underlying cause. PLoS ONE.

[bib0030] Thøfner I.C.N., Dahl J., Christensen J.P. (2021). Keel bone fractures in Danish laying hens: prevalence and risk factors. PLoS ONE.

[bib0031] Thøfner I.C.N., Poulsen L.L., Bisgaard M., Christensen H., Olsen R.H., Christensen J.P. (2019). Correlation between footpad lesions and systemic bacterial infections in broiler breeders. Vet Res.

[bib0032] Tiemann I., Hillemacher S., Wittmann M. (2020). Are dual-purpose chickens twice as good? Measuring performance and animal welfare throughout the fattening period. Animals.

[bib0033] Tierschutz-Nutztierhaltungsverordnung. 2006 (last revision 2021). Verordnung zum Schutz landwirtschaftlicher Nutztiere und anderer zur erzeugung tierischer Produkte gehaltener Tiere bei ihrer haltung. (In German.).

[bib0034] Toscano M.J., Dunn I.C., Christensen J.-P., Petow S., Kittelsen K., Ulrich R. (2020). Explanations for keel bone fractures in laying hens: are there explanations in addition to elevated egg production?. Poult. Sci..

[bib0035] Webster A.B. (2004). Welfare implications of avian osteoporosis. Poult. Sci..

[bib0036] Wei H., Bi Y., Xin H., Pan L., Liu R., Li X., Li J., Zhang R., Bao J. (2020). Keel fracture changed the behavior and reduced the welfare, production performance, and egg quality in laying hens housed individually in furnished cages. Poult. Sci..

[bib0037] Welfare Quality®. 2019. Welfare Quality® Assessment Protocol for Laying Hens (Version 2.0), Lelystad, Netherlands.

